# Low-volume label-free SARS-CoV-2 detection with the microcavity-based optical fiber sensor

**DOI:** 10.1038/s41598-023-28790-y

**Published:** 2023-01-27

**Authors:** Monika Janik, Tomasz Gabler, Marcin Koba, Mirosława Panasiuk, Yanina Dashkevich, Tomasz Łęga, Agnieszka Dąbrowska, Antonina Naskalska, Sabina Żołędowska, Dawid Nidzworski, Krzysztof Pyrć, Beata Gromadzka, Mateusz Śmietana

**Affiliations:** 1 Institute of Microelectronics and Optoelectronics, Warsaw University of Technology, Koszykowa 75, 00-662 Warszawa, Poland; 2 Department of Metrology and Optoelectronics, Faculty of Electronics, Telecommunications and Informatics, Gdańsk University of Technology, Narutowicza 11/12, 80-233 Gdańsk, Poland; 3 National Institute of Telecommunications, Szachowa 1, 02-894 Warsaw, Poland; 4 Institute of Biotechnology and Molecular Medicine, Kampinoska 25, 80-180 Gdańsk, Poland; 5 NanoExpo®, Kładki 24/54, 80-822 Gdansk, Poland; 6 Virogenetics Laboratory of Virology, Malopolska Centre of Biotechnology, Jagiellonian University, Gronostajowa 7a, 30-387 Krakow, Poland

**Keywords:** Health care, Fibre optics and optical communications, Laser material processing, Optical sensors

## Abstract

Accurate and fast detection of viruses is crucial for controlling outbreaks of many diseases; therefore, to date, numerous sensing systems for their detection have been studied. On top of the performance of these sensing systems, the availability of biorecognition elements specific to especially the new etiological agents is an additional fundamental challenge. Therefore, besides high sensitivity and selectivity, such advantages as the size of the sensor and possibly low volume of analyzed samples are also important, especially at the stage of evaluating the receptor-target interactions in the case of new etiological agents when typically, only tiny amounts of the receptor are available for testing. This work introduces a real-time, highly miniaturized sensing solution based on microcavity in-line Mach–Zehnder interferometer (μIMZI) induced in optical fiber for SARS-CoV-2 virus-like particles detection. The assay is designed to detect conserved regions of the SARS-CoV-2 viral particles in a sample with a volume as small as hundreds of picoliters, reaching the detection limit at the single ng per mL level.

## Introduction

At the end of 2019, a new zoonotic virus was detected in China, which shortly after was identified to be Sarbecovirus–severe acute respiratory syndrome coronavirus 2 (SARS-CoV-2)^1^. The emergence of different variants of SARS-CoV-2 and other threats related to viruses across the globe is worrisome, especially as the mutations acquired by viruses increase its transmission affecting diagnostics and vaccine efficacy^1,2^. Hence, there is an urgent need to develop fast, accurate, and accessible virus diagnostic/detection methods. On the other hand, when a new, unknown etiological agent or a new mutation emerges, finding the suitable receptor allowing its detection is crucial, especially when label-free sensing systems are considered. In the label-free concept, on top of high sensitivity to changes in material properties bound to the sensor's surface, the selectivity of the detection by proper surface functionalization and a receptor must be ensured. However, obtaining biorecognition elements against a new target involves a multiple-step procedure to get high-quality, concentrated, specific material. This process often results in a product's extremely small amounts (small concentrations and volumes). Therefore, new detection platforms, except for providing high sensitivity, should also be highly miniaturized, allowing multiple tests out of low-volume samples.

Usually, the receptor-target analysis is performed using the gold standard analytical methods such as flow cytometry, enzyme-linked immunosorbent assay (ELISA), or plasmonic-based platforms, such as surface plasmon resonance (SPR) systems. However, despite the sensitivity and accuracy, they also have some drawbacks. Except for requiring expensive equipment, an advanced laboratory setup, qualified personnel, and the time-consuming procedure, each method requires hundreds of microliters of sample to perform a single test.

Having these issues in mind, during the last years, many label-free, low-volume sensing systems/platforms for virus detection have been developed^3,4^. Among them, we can list electrochemical^5^, plasmonic^6^, nanomaterial-based^6–8^, optical^9^ or microfluidic sensors^10^. However, when considering miniaturized sensors, sensing concepts induced in an optical fiber deserve particular attention. The development of precise microfabrication techniques, such as femtosecond laser ablation, allows for the development of in-fiber interferometric structures such as microcavity in-line Mach–Zehnder interferometer (µIMZI)^11^. The sensor has been described in many different shapes and sizes, such as V-or U-shape^12^, where the diameter of the in-fiber microcavities ranges from 40 to 100 µm^13^. In this sensing scheme, the cavity micromachined in a fiber cladding and partially in the core is a defining feature of the sensor which splits incoming light into two parts – one propagates inside the fiber core (reference beam), and the other propagates through the cavity (sensing beam). The interference of these two beams at the distant sidewall results in an interference pattern. Such a structure not only facilitates the analysis of liquid samples of picoliter volumes but also creates an efficient light-analyte coupling setup that provides one of the highest sensitivities to refractive index (RI) changes in the cavity volume reaching tens of thousands nm/RIU^14^. The combination of advantages of an optical fiber, e.g., rapid measurements and suitability for on-site detection, which may significantly improve user security, with the ability to analyze samples of picoliter volume make them ideal candidates for biosensing purposes. However, until now, the µIMZI has been utilized only for *E. coli* bacteria detection^15,16^ and real-time monitoring of isothermal rolling circle amplification of DNA^17^. It is worth mentioning that in-fiber microcavity-based sensors have never been used for label-free virus detection or such receptor-target analysis.

As the chosen target—SARS-CoV-2 is highly contagious with rapid person-to-person transmission, the research on it requires special facilities with the necessary biosafety levels. Therefore, virus-like particles (VLPs) may be used for new sensing strategies development. VLPs are non-infectious particles composed of multiple proteins that typically form viral capsids that mimic the native virus. Thus, for the last decades, VLPs were successfully used as a surrogate system to overcome the limitations of working with hazardous pathogens such as Ebola virus, Marburg virus, Norovirus, Human Papillomavirus, Rift Valley Fever virus, Highly Pathogenic Influenza Virus, and other human threats^18^. Successful production of SARS-CoV-2 VLPs has been reported for mammalian and insect cell expression systems showing a strong resemblance to the infectious form of a virus^18–24^.

**Figure 1:**
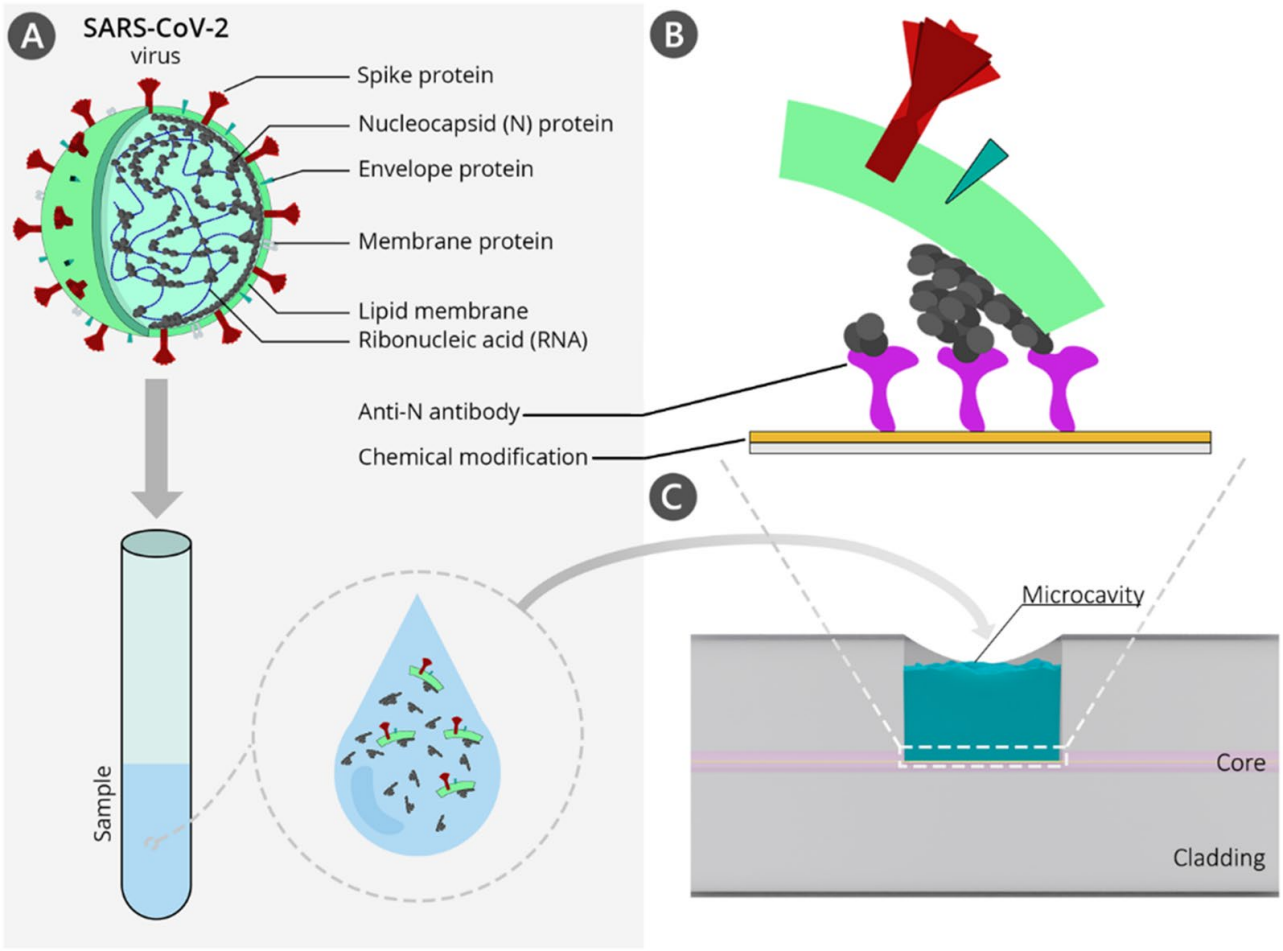
(**A**) Structure of the SARS-CoV-2 virus; (**B**) the detection of nucleoprotein bonded to the elements of SARS-CoV-2 viruses-like particles (VLPs); (**C**) side-view of the µIMZI. Elements are not to scale. *The illustration was created with the use of Adobe Illustrator cc.*

This work reports the first application of a microcavity-based optical fiber sensor for direct SARS-CoV-2 VLPs detection, as it is schematically shown in Fig. 1. Keeping in mind the continuous evolution of the virus, the biosensing approach applied here is based on the anti-nucleocapsid SARS-CoV-2 protein antibodies. N protein is the main component of the virus nucleocapsid and determines the replication cycle through binding to the viral RNA^25^. What is the most important in terms of utilizing protein N as the target, clinical investigations have reported that this protein is expressed in abundance at the early stage of infection^26^. Moreover, in comparison to S protein, which undergoes heavy post-translational modifications, protein N has only one well-characterized o-phosphorylation site at Ser176 and no glycosylation sites; therefore, binding affinity with the receptor is not disturbed, making the protein N a perfect candidate for an early diagnostic target of SARS-CoV-2 infection^25^.

In contrast to other label-free biosensing devices, the work presented here is based on custom-made biological materials which are not commercially available (i.e., SARS-CoV-2 VLPs, anti-protein N (specifically recombinant RNA-binding domain of N protein of SARS-CoV-2) antibodies), and which were obtained in small amounts (volume and concentrations), therefore requiring low-volume sensing system. The sensor presented in this work facilitates measurements of samples of picoliter volumes and offers label-free and real-time monitoring of biomolecular interactions. Therefore, it enables a one-step assay to be performed in not more than 30 min. Applied optimized chemical functionalization of the sensor’s surface allowed to achieve outstanding sensitivity levels in the range of ng per mL. To validate the approach, results received for µIMZI were compared to standard and regulated techniques, such as ELISA.

## Materials and methods

### µIMZI fabrication and preliminary volume sensitivity analysis

Structures in the form of cylindrical cavities with a diameter of 60 µm and depth reaching 62.5 µm were fabricated in a standard Corning SMF-28e fiber through its cladding down to the core using femtosecond laser technology as described in detail in^27^. The µIMZI transmission has been continuously monitored with Leukos SM30 supercontinuum white light source and Yokogawa AQ6370B optical spectrum analyzer in the spectral range 1100–1700 nm. A set of water/glycerin solutions with RI varying in the range of nD = 1.3330–1.3403 RIU was used to perform the reference volume RI sensitivity measurements. The RI of the solutions was measured using Rudolph J57 automatic refractometer and given in RI units (RIU). All the measurements were performed on specially prepared hydrophobic test surfaces, which allowed precise control of the amount of dosed liquid and filling of the microcavity.

### µIMZI surface functionalization

The protocol of the surface functionalization was described in detail in our previous work^16^. Briefly, the silanization process was conducted from a gas phase for 2 h. Next, the silane layer was cured in an argon atmosphere for 48 h. In the next step, the deposited amine groups were activated through immersion in 2.5% glutaraldehyde (GLU) (Sigma-Aldrich) solution in PBS for 30 min. For the conjugation, the sensor was incubated in 0.1 mg/mL anti-N20 Abs solution (in PBS) for 45 min. Finally, Abs immobilization was followed by immersion in 0.5 mg/mL BSA for 30 min to block the non-specific interactions.

### SARS-CoV-2 VLP detection experiment

µIMZI sensor functionalized as described in the previous section was utilized to detect different concentrations of SARS-CoV-2 VLP^21^. To simulate the real clinical sample, the VLPs were kept in the RT for 4 h, and right before incubation of the µIMZI the VLPs dilutions were shaken vigorously, partially destroying the VLPs and releasing protein N. For sensitivity analysis, starting VLPs solutions (300 ng/mL) at dilutions of 100 and 10 in PBS were prepared. For selectivity measurements, negative control solution containing VLPs of norovirus (NoV) was prepared at a concentration of 5000 ng/mL. During the experiments, the sensor was immersed in solutions of increasing concentrations of specific VLPs for 30 min for each concentration. Each step of the experiment was followed by triple extensive washing in PBS. Selectivity measurements were done with µIMZI sensor modified in the same manner with anti-N20 protein Abs and BSA. The sensor was first immersed in non-specific norovirus VLPs and incubated for 30 min, washed, and measured in PBS.

All biological materials utilized within this work namely: RNA-binding domain of nucleocapsid protein of SARS-CoV-2 produced in *E. coli* (N20)—positive control; nucleocapsid protein of NL63 coronavirus produced in *E. coli* (N-NL63)—negative control; anti-nucleocapsid antibodies (Abs)—biorecognition element; and, SARS-CoV-2 virus-like particles (VLPs)—positive control have been produced, purified, and characterized in house. All procedures considering the synthesis of biological materials utilized within these studies have been described in Supplementary Information.

### Target-receptor interaction analysis

#### Titration of anti-N20 antibodies using ELISA assay

The titration of anti-N20 antibodies was conducted similarly to the one reported in^28^. A 96-well ELISA plate (Greiner Microlon High-Binding, clear) was coated with 100 ng/well of recombinant RNA-binding domain of N protein of SARS-CoV-2. N protein of NL63 coronavirus produced in *E. coli* was used as a negative control. The coated plate was incubated overnight at 4 °C. Then the plate was washed 4 × 5 min with 200 µl/well of washing buffer (Phosphate buffered saline (PBS)/0.05%Tween20), blocked for 1 h at 37 °C with 250 µl/well of blocking buffer (3% Bovine Serum Albumin (BSA)/PBS/0.05%Tween20) and washed again as described above. Then 100 µl/well of serial dilutions of rabbit anti-N20 Abs were added and incubated for 1 h at 37 °C. The plate was washed again as previously. Then 100 µl/well of a Peroxidase-conjugated AffiniPure Goat Anti-Rabbit Abs (Jackson Immuno Research) (1:2000 in 3% BSA/PBS/0.05%Tween20) was added and incubated for 1 h at room temperature. Finally, following the last plate-washing step (6 × 5 min with 200 µl/well) 100 µl/well of Horseradish Peroxidase (HRP)-substrate solution was added (1-Step Turbo TMB-ELISA, Thermo Scientific). The plate was incubated in the dark until the blue color developed and stopped by adding 50 µL of 0.5 M sulfuric acid to each well. Absorption was measured at wavelength of 450 nm using a plate reader (TECAN).

#### Titration of recombinant nucleoprotein N20 using ELISA assay

The titration of recombinant nucleoprotein N20 was conducted similarly to the one reported in^28^. A 96-well ELISA plate (Greiner Microlon High-Binding, clear) was coated with serial dilutions of recombinant N protein produced in a bacterial expression system. The coated plate was incubated overnight at 4 °C. Then the plate was washed 4 × 5 min with 200 µl/well of washing buffer (PBS/0.05%Tween20) and blocked for 1 h at 37 °C with 250 µl/well of blocking buffer (3%BSA/PBS/0.05%Tween20), and the plate was washed as previously. Then 100 µl/well of rabbit anti-N20 Abs was added, incubated for 1 h at 37 °C and the plate was washed as previously. Then 100 µl/well of Peroxidase-conjugated AffiniPure Goat Anti-Rabbit Abs (Jackson Immuno Research) (1:2000 in 3% BSA/PBS/0.05%Tween20) was added, incubated for 1 h at room temperature. Finally, following the last plate-washing step (6 × 5 min with 200 µl/well) 100 µl/well of HRP-substrate solution was added (1-Step Turbo TMB-ELISA, Thermo Scientific). The plate was incubated in the dark until the blue color developed and stopped by adding 50 µL of 0.5 M sulfuric acid to each well. Absorption was measured at wavelength of 450 nm using a plate reader (TECAN).

#### Cross-reactivity testing using ELISA test

Cross-reactivity tests were conducted similarly to the one reported in^28^. A 96-well ELISA plate (Greiner Microlon High-Binding, clear) was coated with 100 ng/well of: recombinant N protein produced in a bacterial expression system (N20), recombinant N protein produced in a bacterial expression system (NL63), the full length of the N protein of SARS-CoV-2 produced in insect cells; inactivated upper respiratory track viruses such as Epstein-Barr Virus (EBV), Influenza A virus (IVA), Influenza B virus (IVB), respiratory syncytial virus (RSV), human Norovirus (hNoV). The coated plate was incubated overnight at 4 °C. Then the plate was washed 4 × 5 min with 200 µl/well of washing buffer (PBS/0.05%Tween20), blocked for 1 h at 37 °C with 250 µl/well of blocking buffer (3%BSA/PBS/0.05%Tween20) and the plate was washed as previously. Then 100 µl/well of rabbit anti-N20 Abs was added, incubated for 1 h at 37 °C and the plate washed as previously. Then 100 µl/well of Peroxidase-conjugated AffiniPure Goat Anti-Rabbit Abs (Jackson Immuno Research) (1:2000 in 3% BSA/PBS/0,05%Tween20) was added and incubated for 1 h at room temperature. Finally, following the last plate-washing step (6 × 5 min with 200 µl/well) 100 µl/well of HRP-substrate solution was added (1-Step Turbo TMB-ELISA, Thermo Scientific). The plate was incubated in the dark until the blue color developed and stopped by adding 50 µL of 0.5 M sulfuric acid to each well. Absorption was measured at wavelength of 450 nm using a plate reader (TECAN).

## Results and discussion

### μIMZI volume sensitivity analysis

**Figure 2:**
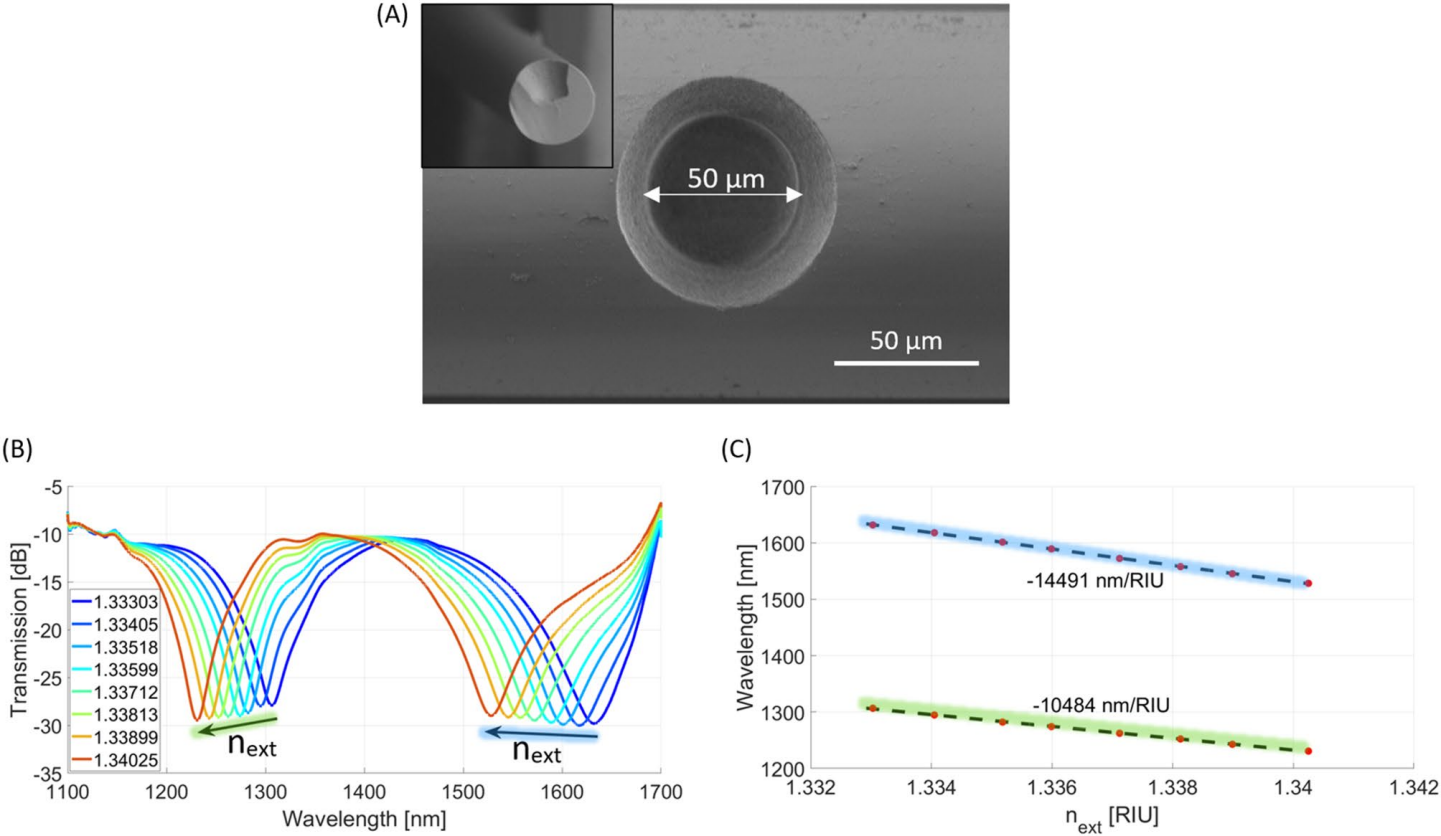
(**A**) SEM images of the µIMZI structure – top view of the structure and its cross section (inset); (**B**) Transmission spectra of μIMZI filled with RI ranging from 1.3333 to 1.3403 RIU and (**C**) corresponding minimum wavelength plotted vs RI – volume sensitivity.

As the sensing performance of the μIMZI differs depending on its structure, having in mind the final application of the sensor, different microcavity designs should be chosen. The crucial parameters to optimize include shape^29^, diameter^30^, and depth of the microcavity^31^. Moreover, washing the microcavity after each step of the experiment–which is of great importance for label-free biosensing–should also be considered^29^. Each μIMZIs utilized in this work were precisely fabricated utilizing the parameters described in Section "µIMZI fabrication and preliminary volume sensitivity analysis". The μIMZI’s structure has been optimized to enable its efficient measurements and cleaning, and, on the other hand, provide mechanical robustness of the sensing system. The exact shape and diameter of the fabricated sensor are shown in Fig. 2A. Furthermore, to characterize the μIMZI sensors before the chemical modification of the surface and the bioassay, the microfabrication process was followed by measurements of the RI sensitivity. Figure 2B presents the transmission spectra of one of the μIMZIs where the RI of the water/glycerol solutions filling the cavity varied in the range from 1.33303 to 1.34025 RIU. As shown in Fig. 2B, both the transmitted power and the minimum’s wavelength change with increasing RI. In Fig. 2C, the corresponding values of the spectral minima are plotted vs. RI. The points corresponding to each sample are linearly approximated with the least-square method identifying the sensitivity in the particular RI region. The μIMZI described in this work displays RI sensitivities ranging from over 10,000 nm/RIU up to over 14,000 nm/RIU for the minimum at shorter (indicated by the green line) and longer wavelengths (indicated by the blue line), respectively (Fig. 2C).

### Characterization and validation of produced target and receptor—target-receptor interaction analysis

All the biological components of the sensing device were validated using in vitro methods such as ELISA assay (Fig. 2).

**Figure 3:**
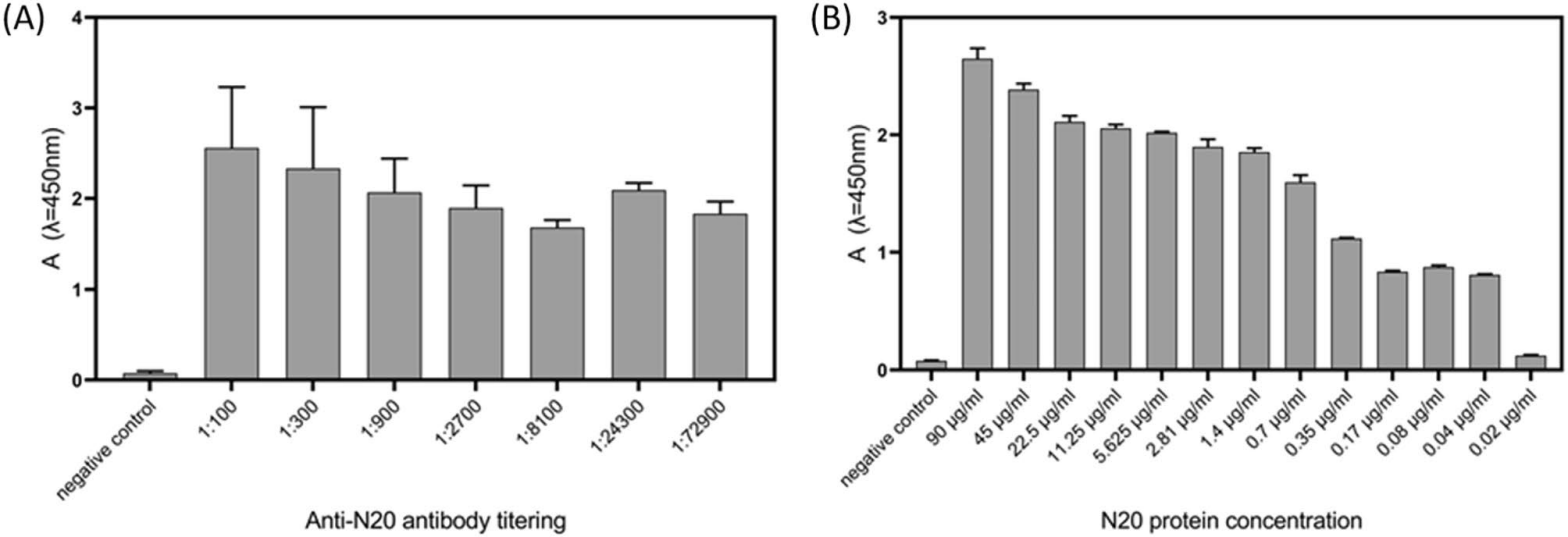
Initial characterization of the biological components for the developed sensing device—µIMZI. (**A**) Titration of the anti-N20 antibodies in ELISA test. (**B**) Titration of protein N20 in ELISA. The nucleocapsid protein from the NL63 coronavirus was used as a negative control.

The obtained truncated form of N protein of SARS-CoV-2 used as a positive control produced in *E. coli* was about 20 kDa (0.27 nm^32^). The full-length N protein of the NL63 coronavirus strain used as a negative control was about 47 kDa. The full-length N protein of SARS-CoV-2 obtained in insect cells was approximately 50 kDa, and finally, the SARS-CoV-2 VLPs obtained in insect cells were approximately 130 nm in size^21^. Produced antibodies (Abs) show great reactivity with the RNA-binding domain of N protein produced in bacteria. Even at the highest dilutions (1:72,900) of Abs (Fig. 3A) and the lowest concentrations (0.04 µg/mL) of protein N (Fig. 3B), the target-receptor interactions are still recognizable.

**Figure 4:**
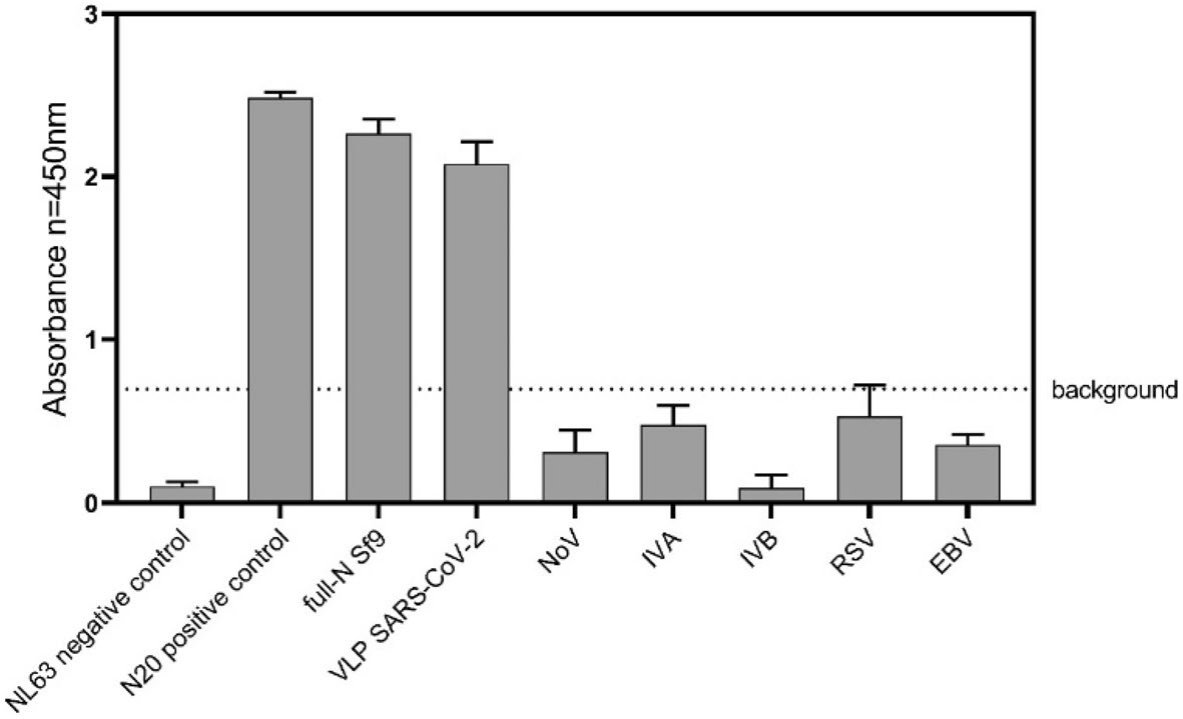
Cross-reactivity of anti-N20 Abs with different upper respiratory tract viruses such as Epstein-Barr Virus (EBV), Influenza A virus (IVA), Influenza B virus (IVB), respiratory syncytial virus (RSV), human Norovirus (hNoV) in comparison to positive controls – N protein, full-length protein N, and SARS-CoV 2 VLPs. Each well was coated with 100 ng of each sample (protein/VLPs/ inactivated virus); Anti-N20 antibodies were used in 1:1000 dilution. The nucleocapsid protein from the NL63 coronavirus was used as a negative control.

Moreover, the specificity of the receptor was extensively tested against different upper respiratory tract viruses, which are often interfering species naturally present in the patients’ samples, such as influenza virus Type A and B, Respiratory Syncytial virus, Ebstain-Barr virus, and human Norovirus (Fig. 4) showing high specificity of the receptor.

### Label-free SARS-CoV-2 VLP detection

For label-free biosensing purposes, the characterized µIMZI (Section "μIMZI volume sensitivity analysis") was first chemically modified with APTES (Section "µIMZI surface functionalization"). Next, glutaraldehyde (GLU) was used to activate the amino groups at the sensor’s surface. Silanization and activation steps were followed by the receptor—the anti-nucleocapsid SARS-CoV-2 protein antibodies – immobilization. Next, 0.5 mg/mL BSA was used to block the surface and avoid any non-specific interactions. Each step of the surface’s modification results in a shift of the minimum towards longer wavelengths (Fig. S1 in Supplementary Information). The direction of the spectral shift is opposite to the one observed during the characterization of the volume sensitivity of the µIMZI (Fig. 2A).

The spectral location of the transmission minimum (*λ*_*m*_) is given by Eq. 1, where *d* is the length (here diameter) of the microcavity, *Δn*_*eff*_ = *n*_*co*_* − n*_*cl*_ is the difference between effective RIs in regions related to the two optical paths, namely *n*_*co*_, and *n*_*cl*_ correspond to the remaining part of the fiber core and micromachined circular cavity, respectively, and *φ*_*0*_ represents an initial phase.

1 $$ \lambda_{m} = \frac{{2\pi d\Delta n_{eff} }}{{\left( {2m + 1} \right)\pi - \varphi_{0} }} $$

Following the analysis reported in^33^, we assumed that the µIMZI response is affected mainly by the effective RI (Δn_eff_), which is the difference between the two interfering modes within the µIMZI, i.e., the one in the remaining part of the core (n_core_) and the one propagating through the cavity (n_cladding_). Altering/influencing each of them results in a different direction of the shift of transmission minimum. Therefore, considering the thickness of the chemical modification layer, as well as the size of the chosen receptor, each step of the surface’s modification affects only the core region (n_core_), and, therefore, results in a shift of the minimum towards longer wavelengths, similarly to the case of the thin-film layer shown in^33^ or modification using peptide aptamers reported in (Janik et al., 2021a).

**Figure 5:**
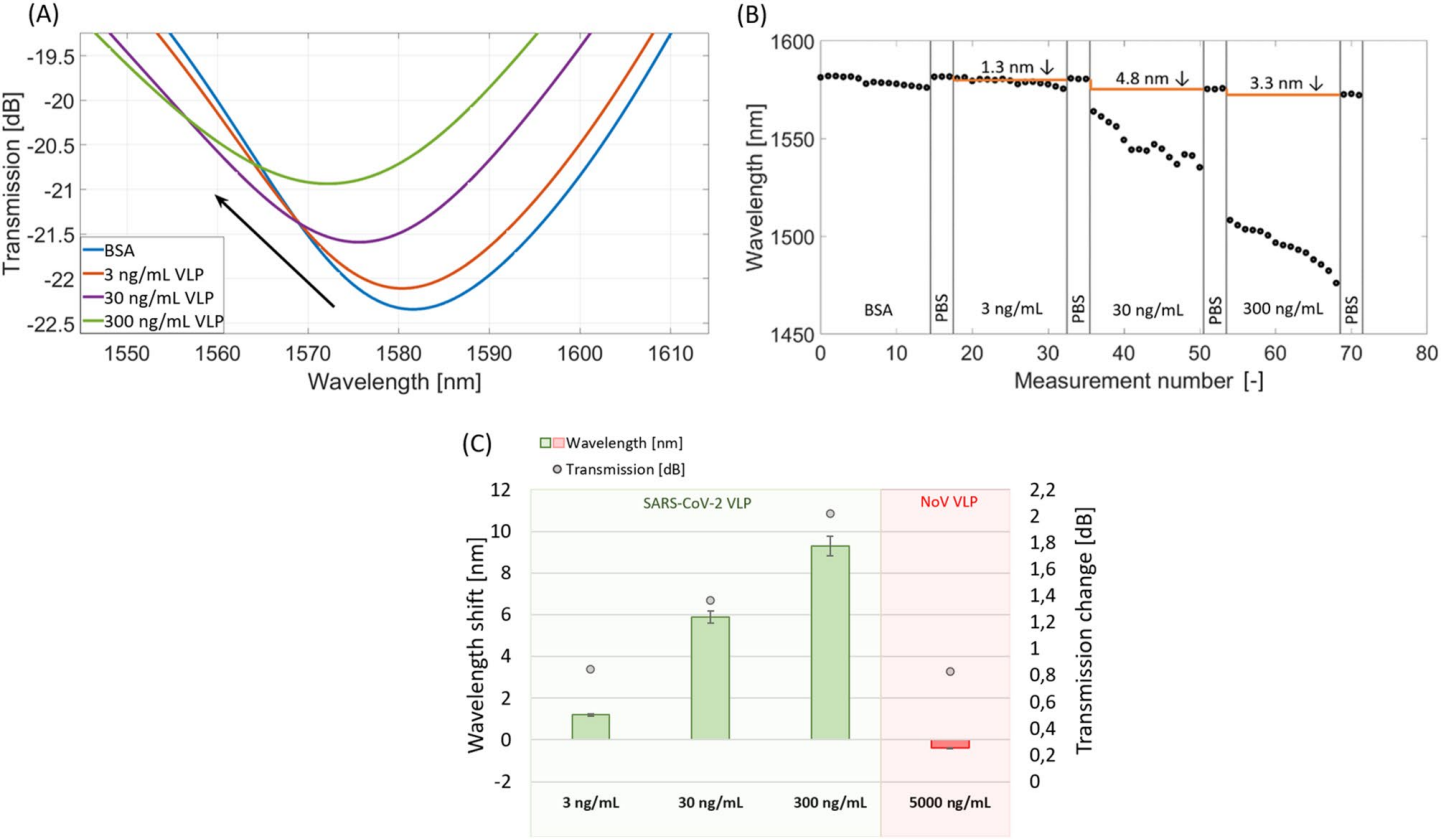
The response of the µIMZI at each stage of the experiment with anti-N Abs used as a receptor and different concentrations of SARS-CoV-2 VLPs. (**A**) The plot shows the transmission spectra in PBS after incubation and extensive washing; (**B**) shows resonance wavelength at subsequent steps of SARS-CoV-2 VLP detection for the followed minimum; (**C**) shows a correlation between the transmission and the wavelength at subsequent steps of SARS-CoV 2 VLPs detection for the followed minimum, as well as for negative control (HNoV VLP). The baseline is indicated with the dashed blue line.

Being aware of the safety issues during the SARS-CoV-2 detection, the following experiments were conducted with the use of the surrogate system of SARS-CoV-2 VLPs produced in Sf9 cells^21^. To our best knowledge, these are the first studies where SARS-CoV-2 VLPs have been used for sensing purposes. The µIMZI sensors modified with antibodies were incubated in solutions with different VLP concentrations ranging from 3 to 300 ng/mL. Figure 5A presents the spectra obtained at each stage of the experiment. Figure 5B presents the wavelength shift measured in a real-time during the experiment.

In general, the higher the concentration of VLPs, the larger the shift of the minimum compared to the baseline. The shift of the followed minimum ranged from 1.3 up to over 10 nm for concentrations 3–300 ng/mL, respectively. Furthermore, changes in the wavelength were followed by an increase in transmission (Fig. 5C). The concentration dependence was repeated on three different µIMZI sensors. Considering the sample treatment, the VLPs’ fractions bonded to the Abs immobilized on the µIMZI’s surface (as shown schematically in Fig. 1) were a combination of protein N alone and protein N bonded/interacting with parts of the virus’s envelope–membrane proteins such as M protein. Because of the size (~ 130 nm) and consecutively rising concentration, the VLPs build up intensively on the sensor’s surface. When such a target is applied, its immobilization on the sensor’s surface, except influencing the mode travelling through the fiber core – n_core_, it may also impact the core travelling through the cavity – n_cladding_. As a result, we expect the shift of the minimum towards shorter wavelengths.

Based on our previous works, we can observe the same effect, i.e., shift towards shorter wavelengths, for increasing RI of liquids within the whole volume of the microcavity, e.g., Fig. 2A, as well as in the case of viruses and bacteria^15,16^ immobilization on the µIMZI’s surface. Although the size of targeting particles may be a reason for the direction of the minimum shift, based on our previous work, the other reason could also be the RI of these parts of the virus. In^15^, the MS2 bacteriophage was immobilized on the µIMZI surface as a bioreceptor for bacteria detection. Its low RI (1.217 RIU) strongly shifted the spectrum towards shorter wavelengths. Unfortunately, the RI of SARS-CoV-2 stays unknown; however, both of described reasons result in the same outcome—the spectrum shift towards shorter wavelengths.

The µIMZI has also been challenged with another surrogate VLPs based on the human pandemic virus causing gastroenteritis—Human Norovirus (HNoV-VLPs) serving as a negative control of the surrogate system. After incubation of the sensor in HNoV (5000 ng/mL) a slight shift (0.82 nm) of the minimum towards longer wavelengths has been recorded (Fig. 5C). Compared to SARS-CoV-2 VLPs where for a ten times lower concentration (300 ng/mL) we obtained over 9 nm shift towards shorter wavelengths it can be stated that in this case, there was no cross-reactivity. However, considering changes in the transmission, it might be caused by the binding of some small parts of the HNoV to the sensor’s surface (as the sample was treated the same as the SARS-CoV-2 VLP—VLPs nanoparticle has been disrupted). These results confirm our previous findings on the selectivity and reliability of the developed sensing concept, where the specificity of the receptor was extensively tested using ELISA assay against different upper respiratory tract viruses (Fig. 3). Based on the above results, the obtained experimental limits of the detection for utilized VLPs were equal to 3 ng/mL.

What is worth mentioning, in parallel, to determine the contribution of the small particles during VLP detection, experiments with the use of the truncated protein N alone have also been conducted. Increasing concentrations of the protein N alone results in small, non-monotonous changes in a wavelength shift (Fig. S2 in Supplementary Information). However, considering the size of the RNA-binding domain of the protein N (~ 20 kDa, ~ 0.27 nm) and the fact that the protein N is over ten times smaller than the receptor placed on the sensor’s surface, the non-monotonous changes could be expected. Based on these results, it can be stated that the protein N alone does not have a crucial impact on the µIMZI response, especially during the detection of the more complex samples such as VLPs.

The obtained level of sensitivity makes the µIMZI better than the ELISA test shown in Fig. 3B, where 10 times higher concentration was statistically insignificant and at a level of the negative control. Furthermore, the level of sensitivity is commensurate with the existing reagent-free lateral flow test that has limits of detection in the ng/mL range^34^. However, it needs to be mentioned that the µIMZI sensor has been designed to detect/target the nucleocapsid protein due to the large copy number of the protein per viral particle (~ 1000)^35^. The common viral loads for COVID-19 positive patients, in turn, are found in the range of 10^5^–10^7^ viruses per mL. Therefore, the N-protein level during infection is significantly higher than the presented LODs. N-protein levels in saliva are in the range of tens of pg/mL at the beginning of COVID-19 to even hundreds of ng/mL within the next days. In blood, these concentrations are smaller but less variable than in saliva and are maintained at levels of tens to hundreds of pg/mL^36^. Therefore, it might be concluded that the µIMZI might be able to diagnose even asymptomatic cases with lower viral loads.

To date, many promising technologies and techniques have been developed for the diagnosis of the SARS-CoV-2 virus. However, there were only a few reported fiber optic-based solutions. The first optical fiber label-free biosensing strategy proposed for the SARS-CoV-2 detection is based on Fiber Optic SPR (FO-SPR)^37^. The sensing concept is built upon the RI change of molecularly imprinted polymers deposited on gold film placed on D-shaped polished fiber, which changes along with the binding of the S1 subunit of protein S. The reported data demonstrate that the sensor can detect the target in various solutions and limit of the detection reached nM of investigated protein. The test was just 10 min, and the sample’s volume needed to conduct the analysis was as low as 50 µL. However, it is noteworthy that the fabrication of the sensor includes a multistep procedure, and the sensing system is based on the SARS-CoV-2 protein S – which is one of the most prone to mutation elements. The same plasmonic-based sensor was also modified with a self-assembled monolayer and selective aptamers to detect the receptor-binding domain (RBD) of the SARS-CoV-2 protein S^38^. In this case, the detection limit was equal to 37 nM. The sample’s volume and time needed to perform the test were the same as in the previous case. SARS-CoV-2 spike protein was also detected using a long-period fiber grating immunosensor^9^. The experimental results revealed that the sensor could detect SARS-CoV-2 spike protein at 100 pg/mL concentration in ~ 20 min. Although the volume needed to perform the experiments was not specified, the sensor's length indicates the volumes' use on the level of hundreds of microliters.

The only fiber-based label-free fiber-based senor targeting the nucleocapsid protein of SARS-CoV-2 is the one reported in (Jia et al., 2021). The experimental results showed that the aptasensor could detect the N protein in nM concentration within 3 min. Knowing that the sensing system is based on the tapered microfiber, the presented analysis can also be considered low-volume. Although the system's performance reached nM concentration of protein N the system had some drawbacks. Based on the authors' analysis, the graphene oxide (GO) layer introduced on the sensor’s surface indeed modified the surface by introducing new functional groups. However, the GO irregularity might also introduce some interruptions or influence the repeatability of the sensor’s fabrication. Moreover, GO, and chosen receptors were immobilized on the surface only through electrostatic interactions, which may be disturbed during the experiment. The authors also reported that aptamers might not completely cover the GO coating. Despite this, no blocking step after receptor immobilization had been introduced, and therefore non-specific interactions could occur.

In this work, µIMZI, compared to the abovementioned sensors, excels in one-step, repeatable fabrication. Moreover, it is universally applicable, making the sensor suitable for detecting any target based on the proper receptor and chemical modification design of the sensor’s surface. Considering the size of the presented sensor and the pL volume analysis, the assay is highly compatible with point-of-care field applications. It may be multiplexed in the future, targeting more than one virus’ protein. This, in turn, could provide additional information on the virus variant/mutation and, therefore, accelerate the decision on the proper treatment choice. It must also be noted that the application of fiber optic for signal transmission enables the separation between a potentially contaminated measurement point from the signal processing setup, which may significantly improve healthcare staff security. The data analyzing device can be placed in a safe and clean localization shielded from contaminated localization.

## Conclusions

In this work, we reported the first microcavity-based fiber optic sensor for virus detection on the example of SARS-CoV-2 VLPs serving as a surrogate system with the use of custom-made biological components. The reported technology allows fast, direct, label-free, specific, and highly sensitive detection of the chosen targets. The lowest detectable value of the demonstrated real-time measurements for the biosensor detecting the SARS-CoV-2 VLP reached single ng/mL. Negative controls and ELISA assay revealed high specificity against different upper respiratory tract viruses, which are often interfering species naturally present in the patients’ samples. What needs to be emphasized, the presented sensor operates at the pL level volume of samples. Therefore, except for detection, the µIMZI could also be utilized to study, analyze and validate the interactions between the low-concentration/low-volume receptors and chosen targets. These, in turn, are critical to both fundamental research, drug discovery, and the development of new sensing platforms, which are crucial, especially at the time of outbreaks, when a new etiological agent, such as SARS-CoV-2 emerges.

## Supplementary Information

Supplementary Information.

## Data Availability

All relevant data is contained within the article. Further inquiries can be directed to the corresponding authors.
