# Supplementary material for: Low-volume label-free SARS-CoV-2 detection with the microcavity-based optical fiber sensor
Source: Sci Rep. 2023 Jan 27;13:1512. doi: 10.1038/s41598-023-28790-y (PMC9880943; doi:10.1038/s41598-023-28790-y)
Supplement: Supplementary file 1 — Supplementary Information. [file 41598_2023_28790_MOESM1_ESM.docx]

Supplementary materials

Article: Low-volume label-free SARS-CoV-2 detection with the microcavity-based optical fiber sensor

Monika Janik^1,2*^, Tomasz Gabler^1^, Marcin Koba^1,3^, Mirosława Panasiuk^4,5^, Yanina Dashkevich^4^, Tomasz Łęga­^4^, Agnieszka Dąbrowska^6^, Antonina Naskalska^6^, Sabina Żołędowska^4^, Dawid Nidzworski^4^, Krzysztof Pyrć^6^, Beata Gromadzka^4,5^, and Mateusz Śmietana^1^

^1^Warsaw University of Technology, Institute of Microelectronics and Optoelectronics, Koszykowa 75, 00-662 Warszawa, Poland

^2^Department of Metrology and Optoelectronics, Faculty of Electronics, Telecommunications and Informatics, Gdańsk University of Technology, Narutowicza 11/12, 80-233 Gdańsk, Poland

^3^National Institute of Telecommunications, Szachowa 1, 02-894 Warsaw, Poland

^4^Institute of Biotechnology and Molecular Medicine, Kampinoska 25, 80-180 Gdańsk, Poland;

^5^NanoExpo®, Kładki 24/54, 80-822 Gdansk, Poland

^6^Virogenetics Laboratory of Virology, Malopolska Centre of Biotechnology, Jagiellonian University,

Gronostajowa 7a, 30-387 Krakow, Poland

**Methodology**

**Biological components**

**1. Production of recombinant proteins**

**1.1. Production of RNA binding domain of nucleocapsid Protein of SARS CoV-2**

The DNA sequence coding RNA -binding domain of N protein of SARS-CoV-2 was synthesized by Thermo Fisher Scientific. Synthetic gene was cloned into the pLATE31 expression vector using ALICator Ligation Independent Cloning (Thermo Fisher Scientific) with primers:

CoV-31F: AGAAGGAGATATAACTATGGGTGGTAGCGGTGGTGGTAGT 
CoV-31R: GTGGTGGTGATGGTGATGGCCGCTACCTTCGGCATAAAAACC.

The recombinant protein was produced in *E. coli* BL21(DE3) by induction with 0,3 mM IPTG at 37°C for 3 h. The cells were harvested by centrifugation and lysed. RNA binding domain of nucleocapsid Protein of SARS CoV-2 was purified under native conditions by Ni-NTA affinity chromatography using HIS-Select® Nickel Affinity Gel [Sigma Aldrich]. The purified recombinant protein was dialyzed to TBS buffer containing 5% glycerol and 10mM EDTA.

**1.2. Production of nucleocapsid Protein of NL63 coronavirus**

The recombinant protein was produced in *E. coli* expression system according to Zuwala et all Plos One 2015.

**1.3. Production of full-length nucleocapsid Protein of SARS-CoV2 in insect cells**

DNA coding sequence of nucleocapsid protein of SARS-CoV-2 with insect enhancer sequence added on the N-end was synthesized by Gene Art Gene Synthesis (Thermo Fisher Scientific). Synthetic sequence was cloned under the polyhedrin promoter into pFastBac1 vector (Invitrogen, Carlsbad, CA) using XhoI and KpnI restriction sites. Recombinant baculovirus was generated according to manufacturer protocol. Briefly: DH10BacTM competent *Escherichia coli* cells were transfected with pFB1-N plasmid. Recombinant bacmid DNA was used to generate recombinant baculovirus (rBV-N) in *Spodoptera frugiperda* (*Sf9*) insect cells. Nucleocapsid protein was produced in suspension culture of *Sf9* cells infected with recombinant baculovirus at an MOI of 3.

**1.4. Antibody production**

Specific polyclonal antibodies against nucleoprotein of SARS-CoV-2 were obtained from a rabbit. A 9-month-old rabbit was immunized twice with 200 μg of RNA -binding domain of N protein of SARS-CoV-2 produced in *E. coli* mixed with Addavax adjuvant. The animal blood was collected 2 weeks after the second injection. IgG rabbit antibodies were purified using Protein A affinity chromatography. Briefly, 15 cm column (Bio Rad) was packed with 3 ml of Protein A-agarose affinity matrix (Roche). The column was pre-equilibrated with 5-bed volumes of Starting buffer (100 mMTris-HCl, pH 8.0 degassed and filtered). 2.0 mL of serum sample was filtered through a 0.2 µm syringe filter to remove insoluble debris. An aliquot of filtered serum (1.0 mL) was mixed with 100 µl of 1.0 M Tris, pH 8.0 to adjust the pH of the crude antibody sample to pH 7.5–8.0. Next, the serum sample was loaded on the protein A-agarose gravity column. The column was washed with 10-bed volumes of washing buffer 1 (100 mMTris-HCl, pH 8.0 degassed and filtered) followed by washing with 10-bed volumes of Washing buffer 2 (10 mMTris-HCl, pH 8.0 degassed and filtered). The rabbit IgGs were eluted using 2-bed volumes of Elution buffer (100 mM glycine, pH 3.0 degassed and filtered). 6 x 1 ml fractions were collected into collection tubes containing 200 µl of Neutralization buffer (1.0 M Tris, pH 8.0). 6 ml of purified rabbit IgG was dialyzed against PBS buffer using Slide-A-Lyzer Dialysis Cassettes (Thermo Scientific). Purified rabbit IgGs were analyzed on SDS-PAGE gel, ELISA, and Western Blott and stored at 4 °C for further use.

**2. Virus-like particles (VLPs) production**

**2.1. SARS-CoV 2 VLPs production**

Nanoparticles surrogate system in form of SARS CoV 2 VLPs were produced as described previously by Naskalska et., al 2021. Brefiely, DNA coding sequence of spike, nucleocapsid protein, envelope protein, and membrane protein of SARS-CoV-2 were synthesized by Gene Art Gene Synthesis (Thermo Fisher Scientific). All synthetics were cloned into pFastBac vectors (Invitrogen, Carlsbad, CA) Recombinant baculoviruses were generated in *Spodoptera frugiperda* (*Sf9*) insect cells. The SARS-CoV 2 VLPs were produced in suspension culture of *Sf9* cells infected with recombinant baculoviruses at an MOI of 5. After 60 h post-infection SARS-CoV2 VLPs were purified from the medium by size exclusion chromatography (SEC).

**2.2 NoV VLPs production**

Purified NoV VLPs were produced in*S. frugiperda* cells as described before in [Janczuk-Richter et., al 2020]. Briefly, the GII.4 NoV 2012 variant (Hu/GII.4/Sydney/NSW0514/2012/AU) capsid DNA coding sequence was synthesized by Gene Art Gene Synthesis (Thermo Fisher Scientific) and cloned into the baculovirus transfer vector pFastBac1 (Invitrogen, Carlsbad, CA) using EcoRI and NotI restriction sites. Recombinant baculovirus rBV-NoV was assembled in *Spodoptera frugiperda* (Sf9) insect cells transfected with bacmid DNA. To produce NoV VLPs, Sf9 cells in suspension culture were infected with rBV-NoV at an MOI of 3. After 60 h post-infection VLPs were purified from medium by size exclusion chromatography (SEC).

**2.3 Inactivated upper respiratory track viruses**

All upper respiratory tract viruses such as Epstein-Barr Virus (EBV), Influenza A virus (IVA), Influenza B virus (IVB), respiratory syncytial virus (RSV) were provided from Sky Way Biolab Company.

3. SARS-CoV-2 protein N detection experiment

The silanized µIMZI sensor with immobilized Abs on its surface was used to detect different concentrations of SARS-CoV-2 N protein. For sensitivity analysis, protein N solutions at concentrations of 1, 10, 100, and 1000 ng/ml in PBS were prepared. During this experiment, the sensor was immersed in the solutions with increasing concentrations of specific protein N for 30 minutes in each concentration. Each step of the experiment was followed by at least triple extensive washing in PBS. The difference between the wavelengths corresponding to the transmission minima after BSA binding and after each exposition to the protein was considered as the sensor response. The concentration dependence was verified using three different µIMZIs.

**Results**

**Chemical modification of the µIMZI’s surface**

Figure S1 presents the transmission spectra after each stage of the µIMZI sensor’s surface functionalization and schematic representation of the surface functionalization process.


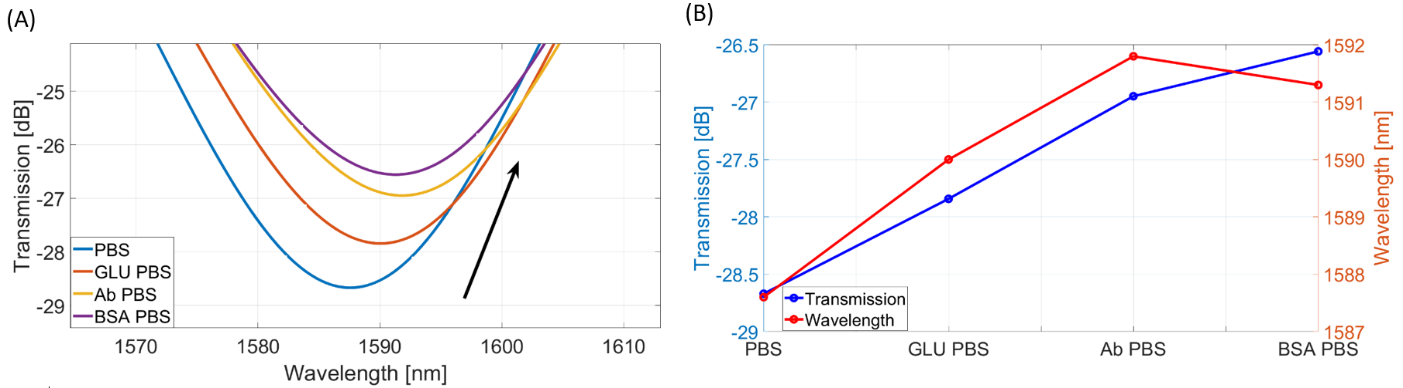


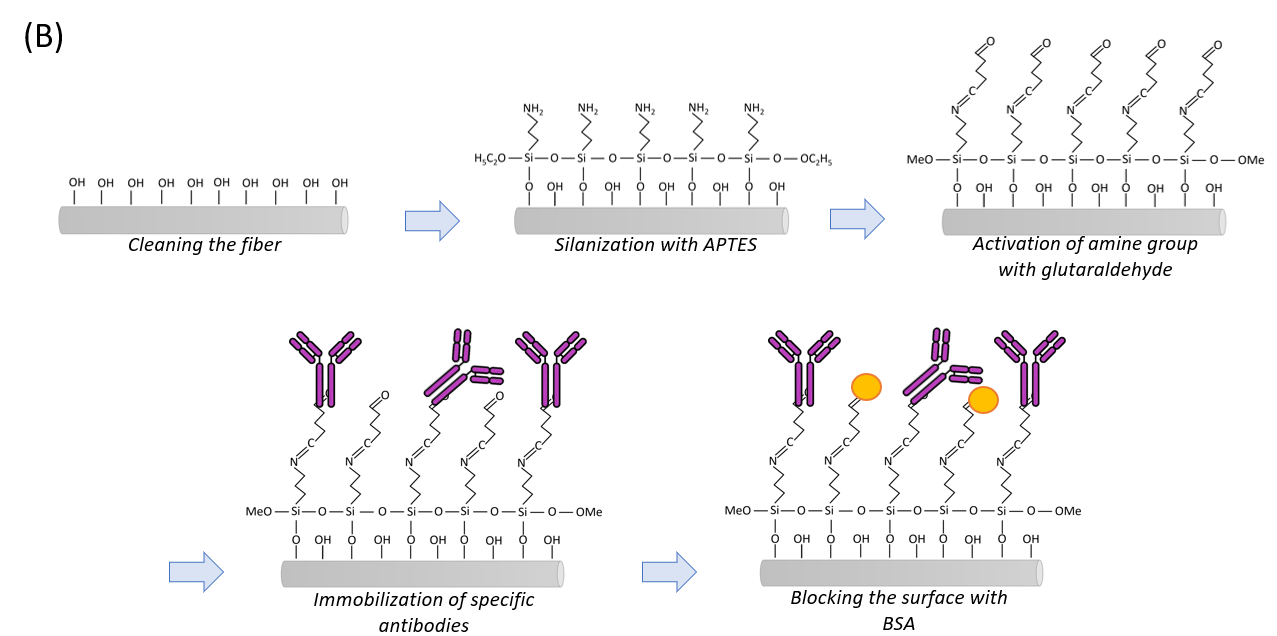


Figure S1 (A) shows the transmission spectra recorded in PBS after each incubation step and extensive washing; (B) shows each stage of the surface functionalization, namely: silanization with APTES, activation with glutaraldehyde (GLU), immobilization of anti-N Abs, and blocking the surface with BSA.

**SARS-CoV-2 nucleocapsid protein detection**

Figure S2 presents the response of the sensor when it is subjected to different protein N concentrations (1 ng/mL to 1 µg/mL).


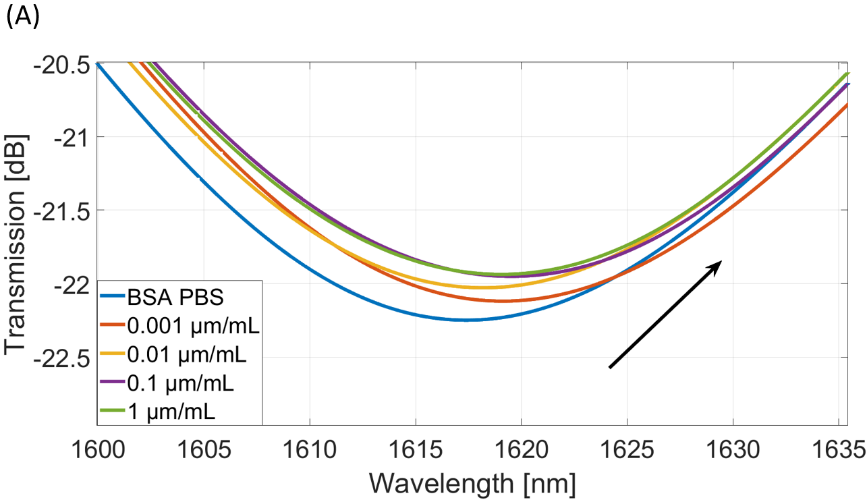


Figure S2. The response of the µIMZI at each stage of the experiment with anti-N20 Abs used as a receptor and different concentrations of SARS-CoV 2 protein N20. A) The plot shows the transmission spectra in PBS after incubation and extensive washing; B) shows a correlation between the transmission and the wavelength; C) shows resonance wavelength at subsequent steps of N20 detection for followed minimum.

Considering the size of the RNA-binding domain of the protein N (~20 kDa, ~0.27 nm), its binding to the Abs immobilized on the sensor’s surface should further influence the *n_core_*, therefore, shifting the spectrum towards longer wavelengths, same as in the case of chemical functionalization (Fig. S1). Indeed, for the lowest concentration of the protein, we can report such a tendency – a 1.7 nm shift towards longer wavelengths – indicating successful detection of the target. However, the second concentration induces a 0.9 nm shift of the minimum to the opposite direction. The third concentration (0.1 µg/mL) induces a 1.3 nm shift again towards longer wavelengths. After incubation with the highest concentration of protein N (1 µg/mL), the sensor revealed 0.5 nm minimum’s shift towards a shorter wavelength, while the step is barely noticeable in terms of the transmission and therefore it can be stated that we are close to the saturation point. Every previous concentration result in an increase in the transmission validating detection of the target.

The effect of changing the shift direction can be caused by the following reason. Based on the chemical modification, which has been utilized in this work, we are expecting, that the Ab is covalently linked to the sensor’s surface most likely via the lysine cluster of the Abs’ heavy chain. Therefore, we expect a relatively flat orientation of the Ab on the surface where one or both heavy chains and one light chain are close to the surface, while the second light chain is directed away from the surface. Such orientation should favour the interaction of the light chain with a few times smaller N protein; however, it will never allow for the creation of an additional uniform layer. Increasing concentrations of the target will be bound as “islands” on the sensor’s surface successively changing the effective RI, which, in turn, results in non-monotonous changes in a wavelength shift. However, they will not be able to create a dense, uniform layer.
